# Supplementary material for: Limited Benefit of Marine Protein Hydrolysate on Physical Function and Strength in Older Adults: A Randomized Controlled Trial
Source: Mar Drugs. 2021 Jan 27;19(2):62. doi: 10.3390/md19020062 (PMC7912527; doi:10.3390/md19020062)
Supplement: Supplementary file 1 [file marinedrugs-19-00062-s001.pdf]

Data Sheet

Marine Peptides

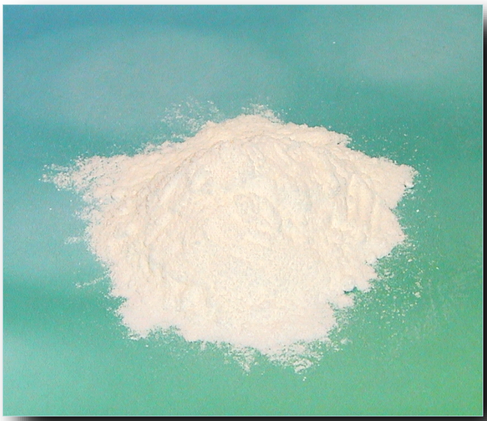

Protein hydrolysate manufactured by hydrolysis of fresh or fresh frozen meat of Atlantic Cod (*Gadus morhua*) using industrial food approved non-GMO proteolytic enzymes. The hydrolysis is performed with equipment and procedures according to regulations given by the Norwegian food authorities. Marine Peptides is approved as food ingredient in Norway according to EU-regulations.

Energy

Kcal / 100 g	362
KJ / 100 g	1514

Proximal composition (%)

Protein (Nx6.25)	> 88.0
Total fat	< 0.2
Carbohydrates	0
Water	< 3.0
Ash	10.0

Minerals (%)

Salt (NaCl)	0.1
Sodium	1,7
Chroride	0.07

Heavy metals (mg/kg)

Lead	< 0.05
Cadmium (Cd)	0.03
Mercury (Hg)	0.11
Arsenic	49.0

Microbiogy (per gram)

Coliform bacteria (37 °C)	0
Termotolerant colif. (44.5 °C)	0
Coagulase pos Staphylococcus	0
Mould and yeast	< 100
Salmonella spp	0
Listeria nonoctogenes	0

Solubility, smell and taste

Easily soluble in cold water giving a clear solution. No bittertaste. Weak sweetish smell and very low note of marine fish; easily masked on demand in applications.

Shelflife

Minimum 3 years i unopened package.

Amino acid profile (mg/g)

Non-essential amino acids

Aspartic acid	96.2
Glutamic acid	157.8
Hydroxyproline	4.8
Serine	42.4
Glycine	48.3
Alanine	63.2
Proline	30.9
Tyrosine	25.4
Arginine	56.9

Essential amino acids

Histidine	28.4
Threonine	37.0
Methionine	27.2
Fenylalanine	27.1
Valine	40.5
Isoleucine	36.4
Leucine	76.2
Lysin	92.5
Tryptofan	5.5

Sum amino acids	888.0
Essential amino acids (EAA)	367
Non-essential amino acids (NEAA)	521
Ratio EAA/NEAA	***0.70***
Branched Chain Amino Acids	153.1
Estimated content of *Glutamine*	78.9

Molecular weight distribution analysis by exclusion chromatography shows that *15 %* of the pep-tides have molecular weights higher than 5.000 Da (more than 36 amino acids in the peptides), *10 %* between 5.000 and 2.500 Da (36 to 18 amino acids in the peptides), *10 %* between 2.500 and 1.000 Da (18 to 7 amino acids in the peptides), and *55 %* with molecular weight less than 1.000 Da (ie 7 or less amino acids in the peptides).
